# Cell cycle-dependent activation of proneural transcription factor expression and reactive gliosis in rat Müller glia

**DOI:** 10.1038/s41598-023-50222-0

**Published:** 2023-12-19

**Authors:** Reiko Nishino, Kaori Nomura-Komoike, Tomohiro Iida, Hiroki Fujieda

**Affiliations:** 1 Department of Anatomy and Neurobiology, School of Medicine, Tokyo Women’s Medical University, 8-1 Kawada-cho, Shinjuku-ku, Tokyo, 162-8666 Japan; 2 Department of Ophthalmology, School of Medicine, Tokyo Women’s Medical University, Tokyo, Japan

**Keywords:** Neuroscience, Stem cells

## Abstract

Retinal Müller glia have a capacity to regenerate neurons in lower vertebrates like zebrafish, but such ability is extremely limited in mammals. In zebrafish, Müller glia proliferate after injury, which promotes their neurogenic reprogramming while inhibiting reactive gliosis. In mammals, however, how the cell cycle affects the fate of Müller glia after injury remains unclear. Here, we focused on the expression of proneural transcription factors, Ngn2 and Ascl1, and a gliosis marker glial fibrillary acidic protein (GFAP) in rat Müller glia after *N*-methyl-*N*-nitrosourea (MNU)-induced photoreceptor injury and analyzed the role of Müller glia proliferation in the regulation of their expression using retinal explant cultures. Thymidine-induced G1/S arrest of Müller glia proliferation significantly hampered the expression of Ascl1, Ngn2, and GFAP, and release from the arrest induced their upregulation. The migration of Müller glia nuclei into the outer nuclear layer was also shown to be cell cycle-dependent. These data suggest that, unlike the situation in zebrafish, cell cycle progression of Müller glia in mammals promotes both neurogenic reprogramming and reactive gliosis, which may be one of the mechanisms underlying the limited regenerative capacity of the mammalian retina.

## Introduction

Müller glia, the principal glial cells in the retina, have a capacity to regenerate neurons in response to retinal injury. However, such regenerative capacity of Müller glia varies widely across species. In zebrafish, injury induces Müller glia to proliferate and dedifferentiate to highly proliferative neuronal progenitors that are capable of regenerating lost neurons^1^. In mammals, however, the neurogenic potential of Müller glia is extremely limited and they undergo reactive gliosis instead of neurogenesis after injury^2^. Mouse Müller glia can be stimulated to proliferate by exogenous growth factors^3,4^ or Hippo pathway inactivation^5,6^. Also, forced expression of proneural factors such as Ascl1 induces neurogenic potential of mouse Müller glia^7–9^. However, retinal regeneration in mammals remains extremely challenging due, at least in part, to the presence of endogenous mechanisms restricting neurogenic reprogramming of Müller glia^10,11^.

Proliferation of Müller glia is essential for retinal regeneration in zebrafish^12,13^. However, it remains unclear whether proliferation has any role in the injury-induced regenerative responses of mammalian Müller glia. The proliferative ability of mammalian Müller glia varies between species. Mouse Müller glia rarely proliferate after injury while rat Müller glia are capable of proliferation in a variety of injury models^14–16^. Injury triggers the expression of cyclin D1, the G1 phase regulator, in Müller glia of both mice and rats; however, the upregulation of cyclin D1 is much more robust in rats compared to mice and the expression of cyclin E and A, essential for G1/S transition, is activated only in rats^16,17^. The mechanism underlying the species difference in the proliferative ability of Müller glia remains unclear, but we have recently shown that phosphatidylserine and Rac1, key regulators of phagocytosis, are involved in the injury-induced proliferation of rat Müller glia^18^. We have further reported that mouse Müller glia, when forced to proliferate by dissociation culture, upregulate reprogramming factors Pax6 and Vsx2 as well as a gliogenic factor Nfia during cell cycle progression^17^. This indicates that cell cycle progression may influence the fate of Müller glia after injury through the regulation of lineage-specific transcription factor expression; however, experimental evidence that the cell cycle regulates the expression of lineage-specific factors in mammalian Müller glia is lacking.

In the present study, we took advantage of the rat model of *N*-methyl-*N*-nitrosourea (MNU)-induced photoreceptor injury^16,18^ to investigate the role of cell cycle progression in the regulation of neurogenic or reactive responses of Müller glia. By reversibly inducing G1/S cell cycle arrest by thymidine block, we demonstrated that S phase entry of Müller glia promotes the expression of proneural factors Ascl1 and Ngn2 as well as a gliosis marker glial fibrillary acidic protein (GFAP). Our data suggest that proliferation of mammalian Müller glia may have both neurogenic and antineurogenic effects highlighting the importance of defining how the cell cycle impacts the fate of Müller glia after injury.

## Results

### Expression of cell cycle markers and proneural factor Ngn2 in Müller glia after photoreceptor injury in vivo

**Figure 1:**
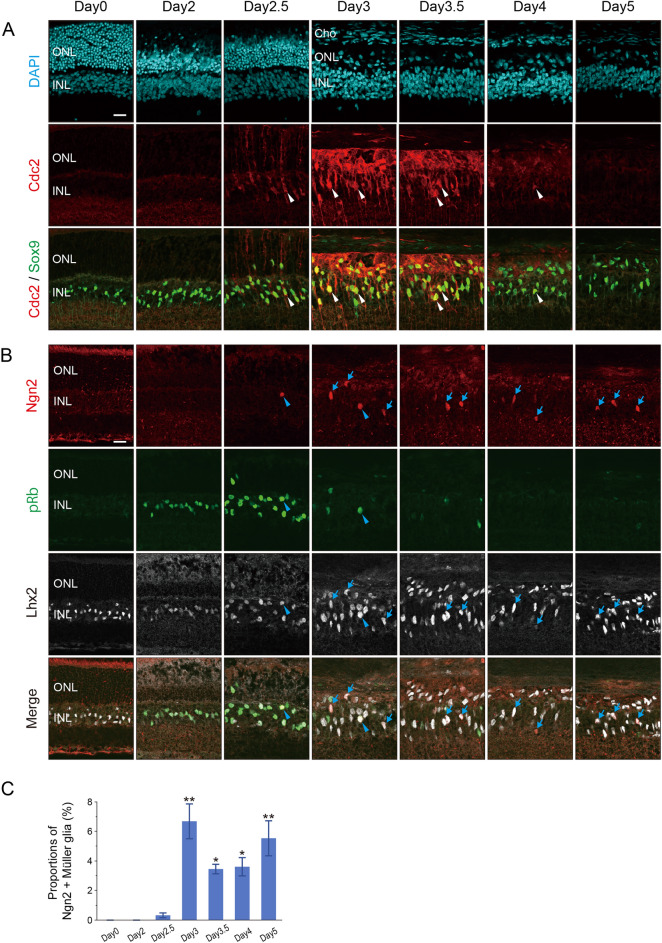
Expression of Cdc2 and Ngn2 in Müller glia of the rat retina after MNU-induced photoreceptor injury. (**A**) Double immunofluorescence for Cdc2 and Sox9 (Müller glia marker). DAPI staining shows the retinal layers. Arrowheads indicate colocalization. (**B**) Triple immunofluorescence for Ngn2, pRb, and Lhx2 (Müller glia marker). Arrowheads indicate Ngn2/pRb double-positive Müller glia while arrows denote Ngn2-positive/pRb-negative Müller glia. (**C**) Quantification of Ngn2-positive Müller glia. Each bar represents the mean ± SEM (n = 3). *P < 0.05, **P < 0.01 (in comparison to Day 0). ONL, outer nuclear layer; INL, inner nuclear layer. Cho, Choroid. Scale bar = 20 µm. The scale bar shown in the upper left corner image applies to all images in the same panel.

We generated a rat model of photoreceptor injury by an intraperitoneal injection of MNU as reported previously^16^. Consistent with the previous observations^16^, the outer nuclear layer (ONL) of the retina became TUNEL-positive by day 2 after MNU treatment and most of the TUNEL-positive photoreceptors were subsequently removed by day 3 (Supplementary Fig. S1A). As we planned to induce G1/S cell cycle arrest of Müller glia in retinal explants by thymidine block, we searched for a cell cycle marker suitable to verify G1/S arrest. Because thymidine analogues, such as BrdU and EdU, commonly used to label S phase, cannot be used in thymidine experiments, we tested Cdc2 (Cdk1), a member of the cyclin-dependent kinase (CDK) family known to regulate the G2/M transition^19^. Based on its function, we hypothesized that Cdc2 would be expressed in proliferating Müller glia after S phase entry. We thus conducted double immunofluorescence for Cdc2 and Sox9 (Müller glia marker) in the MNU-treated rat retinas. Cdc2 was not detectable in the control retinas (day 0) and at day 2, when most Müller glia are in G1 phase as shown previously^16^ (Fig. 1A). Cdc2 was first detected in the nuclei as well as the cytoplasm of Müller glia at day 2.5 (Fig. 1A), the timing when most Müller glia are in S phase^16^. Many Sox9-positive Müller glia nuclei were dislocated to the ONL by day 3, and Cdc2 labeling significantly increased in intensity at day 3 and 3.5, but decreased to low levels by day 4 (Fig. 1A). As most Müller glia exit the cell cycle by day 3.5^16^, the presence of Cdc2 labeling at day 3.5 and 4 indicates that Müller glia express Cdc2 for a short period after cell cycle exit although the possibility cannot be excluded that a few Cdc2-positive Müller glia were still in the cell cycle. We thus concluded that Cdc2 is upregulated in Müller glia during S phase and downregulated after cell cycle exit. The presence of Cdc2 in the cytoplasm as well as in the nuclei of Müller glia is consistent with the previous report that the Cdc2/cyclin B complex is cytoplasmic in interphase and is translocated into the nucleus in M phase^20^.

We next examined the expression of Ngn2, a proneural transcription factor, in Müller glia after photoreceptor injury. We conducted triple immunofluorescence for Ngn2, phospho-pRb (pRb, a pan-cell cycle marker), and Lhx2 (Müller glia marker). Immunofluorescence for pRb revealed that most Müller glia reenter the cell cycle by day 2 and exit the cell cycle by day 3.5 (Fig. 1B), confirming our previous report^16^. Ngn2 immunoreactivity was absent in the control retinas and at day 2, but a few Ngn2-positive Müller glia, which were mostly pRb-positive, were found at day 2.5 (Fig. 1B,C). The proportion of Ngn2-positive Müller glia increased to approximately 7% by day 3, but did not change significantly after this stage. Only a small fraction of Ngn2-positive Müller glia (12.5 ± 1.0%, n = 3) were pRb-positive at day 3 (Fig. 1B). That most Ngn2-positive Müller glia were pRb-positive at day 2.5 and became pRb-negative by day 3 indicates that Ngn2 is expressed in Müller glia after S phase entry and retained after cell cycle exit.

### The effects of thymidine block on the cell cycle progression of Müller glia after photoreceptor injury

**Figure 2:**
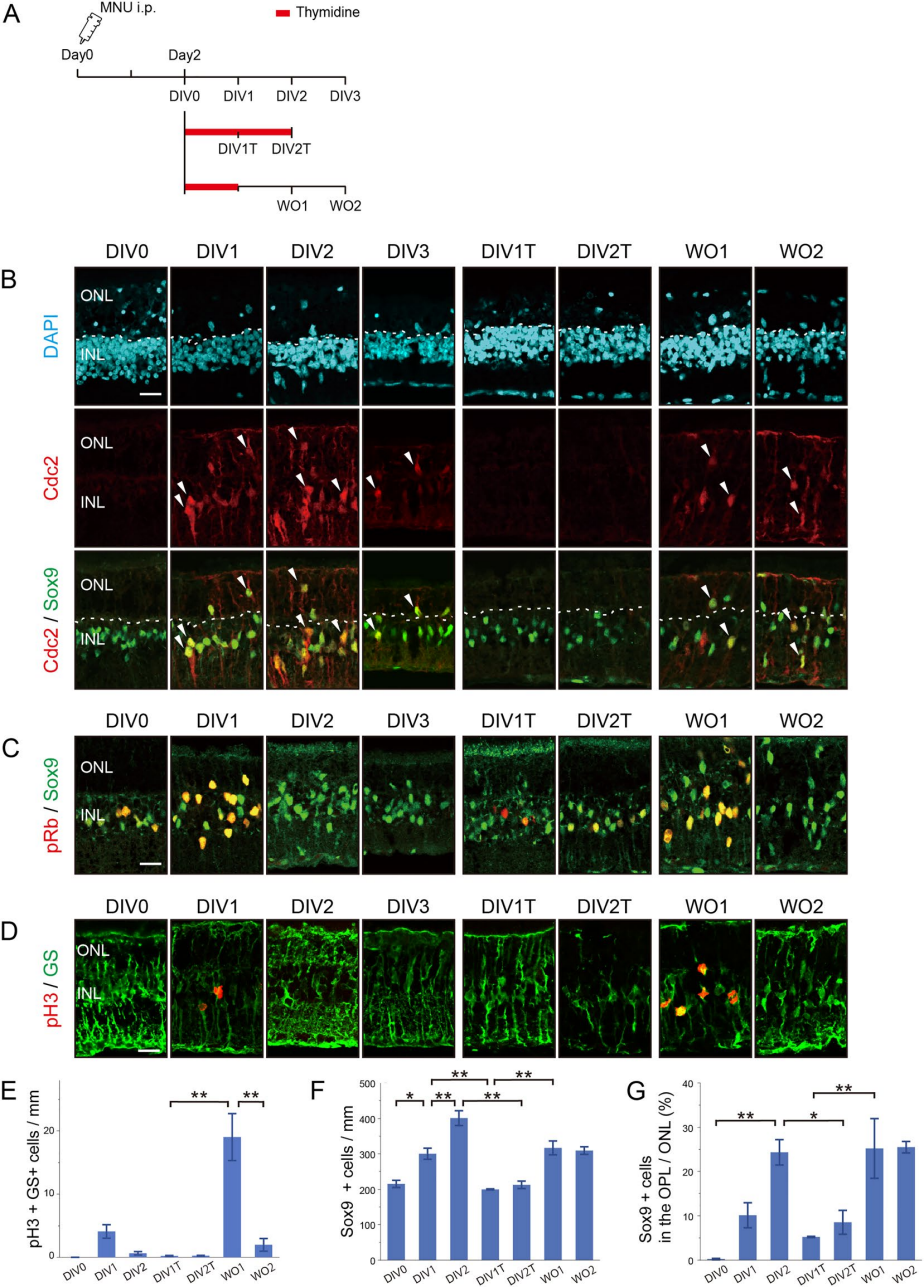
Thymidine-induced cell cycle arrest of Müller glia in retinal explants. (**A**) Experimental design. Retinas were dissected two days after MNU treatment and explant-cultured with and without thymidine for indicated periods. (**B**) Double immunofluorescence for Cdc2 and Sox9. Arrowheads indicate colocalization. Dotted lines indicate the outer margin of the INL. (**C**) Double immunofluorescence for pRb and Sox9. (**D**) Double immunofluorescence for pH3 and glutamine synthetase (GS, Müller glia marker). (**E**) Density of pH3/GS-double-positive Müller glia. (**F**) Density of Sox9-positive Müller glia. (**G**) Proportions of Sox9-positive Müller glia located in the OPL/ONL. Sox9-positive nuclei located outside the dotted lines shown in (**B**) were counted as dislocated Müller glia. DIV, days in vitro; WO, days after wash out; ONL, outer nuclear layer; INL, inner nuclear layer. Scale bars in (**B**), (**C**), and (**D**) = 20 µm. Each bar of the graphs represents the mean ± SEM (n = 3). *P < 0.05, **P < 0.01.

The present study was aimed to elucidate the role of the cell cycle in the regulation of Müller glia responses after injury. To this end, we explant-cultured the rat retinas 2 days after MNU treatment, when most Müller glia are in G1 phase, and analyzed the effects of thymidine-induced cell cycle arrest at the G1/S boundary (Fig. 2A). We first tested whether explant culturing and/or thymidine treatment affect the survival of Müller glia or retinal neurons. TUNEL assays in combination with Sox9 immunofluorescence revealed that TUNEL-positive Müller glia were virtually absent in the retinal explants with and without thymidine treatment (Supplementary Fig. S1B). In contrast, TUNEL/DAPI double-positive nuclei (Sox9-negative) slightly increased from DIV0 to DIV1, and thymidine treatment further increased TUNEL-positive cell number (DIV1 vs. DIV1T) (Supplementary Fig. S1B,C). However, because thymidine treatment for 2 days did not increase TUNEL-positive cell number (DIV2 vs DIV2T) and the numbers of TUNEL-positive cells were very small (20–40 cells/mm retina), we concluded that the effects of explant culturing and thymidine treatment on retinal cell survival are negligible at least for the experimental period examined.

We next characterized Müller glia proliferation in retinal explants with and without thymidine treatment. The previous report indicated that S phase entry of Müller glia peaked at DIV1 (corresponding to day3 in vivo) in the same explant model^18^. Cdc2 was detected in many Müller glia at DIV1 and DIV2 but decreased by DIV3 (Fig. 2B). The absence of pRb and phospho-histone H3 (pH3, M phase marker) at DIV2 and after indicates that most Müller glia exit the cell cycle by DIV2 (corresponding to day 4 in vivo) (Fig. 2C–E). These results suggest that Cdc2 is upregulated in Müller glia after S phase entry (DIV1) and retained for a short period after cell cycle exit (DIV2 and DIV3), consistent with the pattern of Cdc2 expression in vivo. As thymidine blocks the cell cycle at the G1/S boundary, we expected that Müller glia would be arrested in G1 phase by thymidine treatment. In the presence of thymidine (DIV1T and DIV2T), immunoreactivity for Cdc2 and pH3 was lost (Fig. 2B,D,E) indicating that thymidine treatment blocked S phase entry and subsequent cell cycle progression of Müller glia as expected. The presence of pRb-positive Müller glia at DIV1T and DIV2T suggests that some Müller glia remain in G1 phase after thymidine treatment (Fig. 2C). Release from thymidine block (WO1) allowed S phase entry and subsequent cell cycle progression of Müller glia as shown by increase in Cdc2, pRb, and pH3 immunoreactivity (Fig. 2B–E). Loss of pRb and pH3 by 2 days after release (WO2) indicates that most Müller glia exit the cell cycle by WO2 (Fig. 2C–E). We also examined the expression of p27 and cyclin D3, cell cycle regulators expressed in quiescent Müller glia^16,17^. We previously reported that Müller glia lose p27 immunoreactivity after G1 phase entry and re-express p27 after cell cycle exit^16^. Consistent with this report, most Müller glia were p27-negative at DIV0 and DIV1, and became p27-positive at DIV2 (Supplementary Fig. S2A). Many Müller glia remained p27-negative after thymidine treatment (DIV1T and DIV2T), indicating that they were arrested in G1 phase. Most Müller glia became p27-positive at WO2 consistent with their cell cycle exit (Supplementary Fig. S2A). Cyclin D3 immunoreactivity in Müller glia was relatively weak at DIV0 and DIV1, but notably increased after thymidine treatment (DIV1T and DIV2T) (Supplementary Fig. S2B). Release from thymidine block (WO1 and WO2) resulted in a drastic reduction in cyclin D3 immunoreactivity (Supplementary Fig. S2B).

We also counted the number of Sox9-positive Müller glia with and without thymidine treatment. The number of Müller glia increased by approximately two folds by DIV2 in the absence of thymidine, but no significant increase was observed after thymidine treatment (DIV1T and DIV2T) (Fig. 2F). Müller glia number increased significantly after release from thymidine block (Fig. 2F). Furthermore, we analyzed the effects of thymidine treatment on the migration of Müller glia after injury. The proportion of Sox9-positive Müller glia dislocated in the outer plexiform layer (OPL) or ONL increased significantly by DIV2 (Fig. 2G). The migration of Müller glia was inhibited by thymidine and stimulated by release from thymidine block (Fig. 2G), indicating that S phase entry of Müller glia promotes their migration to the ONL.

**Figure 3:**
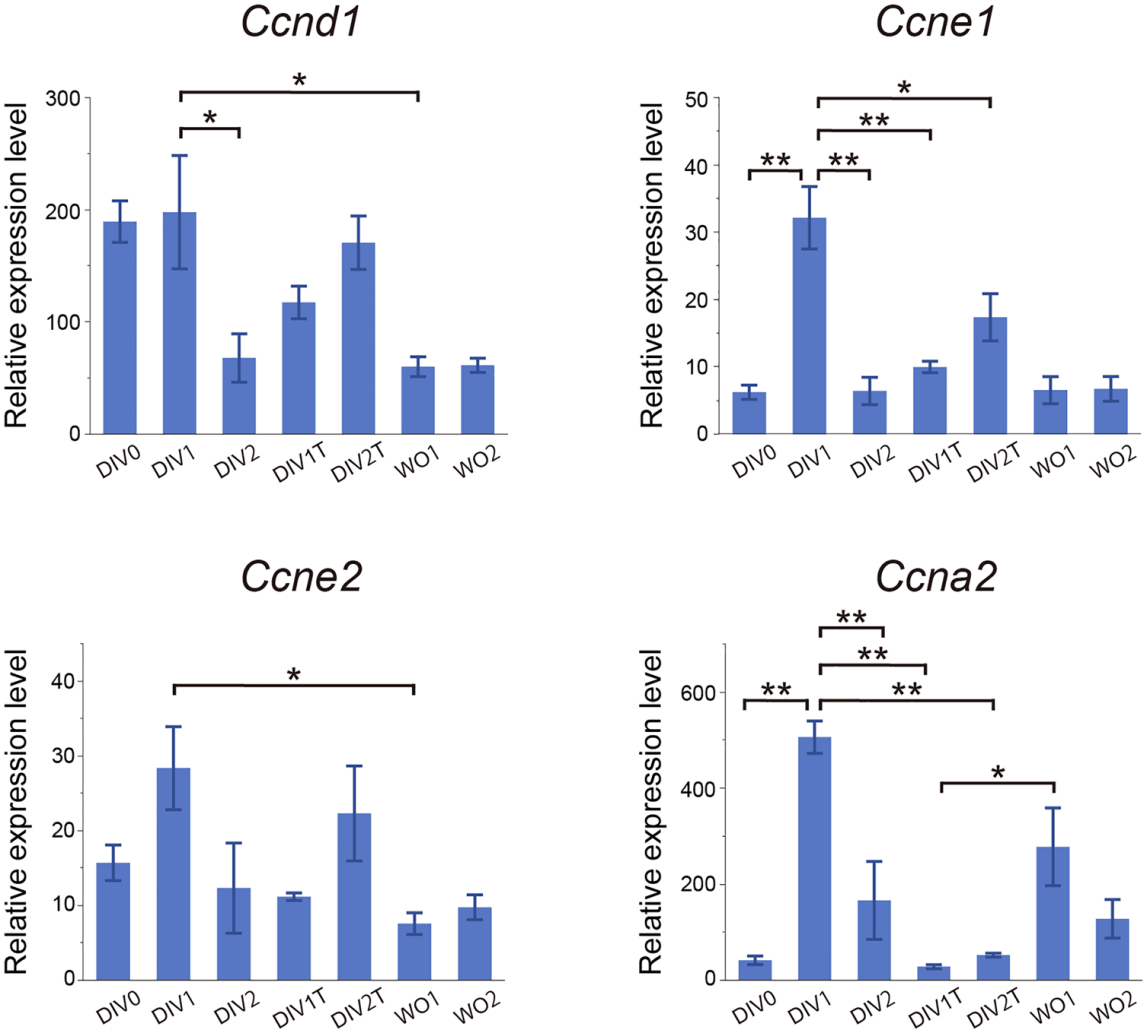
qRT-PCR analyses of the effects of thymidine treatment on the expression of cell cycle genes. DIV, days in vitro; WO, days after wash out. Each bar represents the mean ± SEM (n = 3) with the values expressed relative to the controls (day0 in vivo) after normalization to *Gapdh* levels. *P < 0.05, **P < 0.01.

We further analyzed the expression of the cyclin genes in retinal explants by quantitative RT-PCR (qRT-PCR). We have previously shown that Müller glia, but not other cell types, proliferate in the MNU-treated rat retinas in vivo^16^ or retinal explants^18^ at least for the experimental period examined and that qRT-PCR of the cyclin genes well reflect cell cycle progression of Müller glia^16^. The expression of cyclin D1 (*Ccnd1*), the regulator of G1 phase, was dramatically increased by DIV0 (corresponding to G1 phase) compared to in vivo controls, did not change significantly from DIV0 to DIV1 (S phase entry), and significantly decreased from DIV1 to DIV2, consistent with cell cycle exit of Müller glia (Fig. 3). The expression of cyclin E1 (*Ccne1*) and cyclin A2 (*Ccna2*), the regulator of G1/S transition and S phase progression, respectively, showed significant increase from DIV0 to DIV1 (S phase entry) and significant decrease from DIV1 to DIV2 (cell cycle exit) (Fig. 3). Thymidine treatment significantly suppressed the expression cyclin E1 and cyclin A2, but not cyclin D1 (DIV1 vs DIV1T/DIV2T), consistent with G1 arrest (Fig. 3). Release from thymidine block significantly increased cyclin A2 but did not affect the cyclin D1 and cyclin E1 levels (DIV1T vs WO1) (Fig. 3). The expression of cyclin E2 (*Ccne2*) did not show notable changes by the treatments. Collectively, these data indicate that the expression of the cyclin genes faithfully reflects the cell cycle progression of Müller glia and the effects of thymidine treatment observed by histological analyses.

### The effects of thymidine block on the expression of proneural transcription factors and GFAP after photoreceptor injury

**Figure 4:**
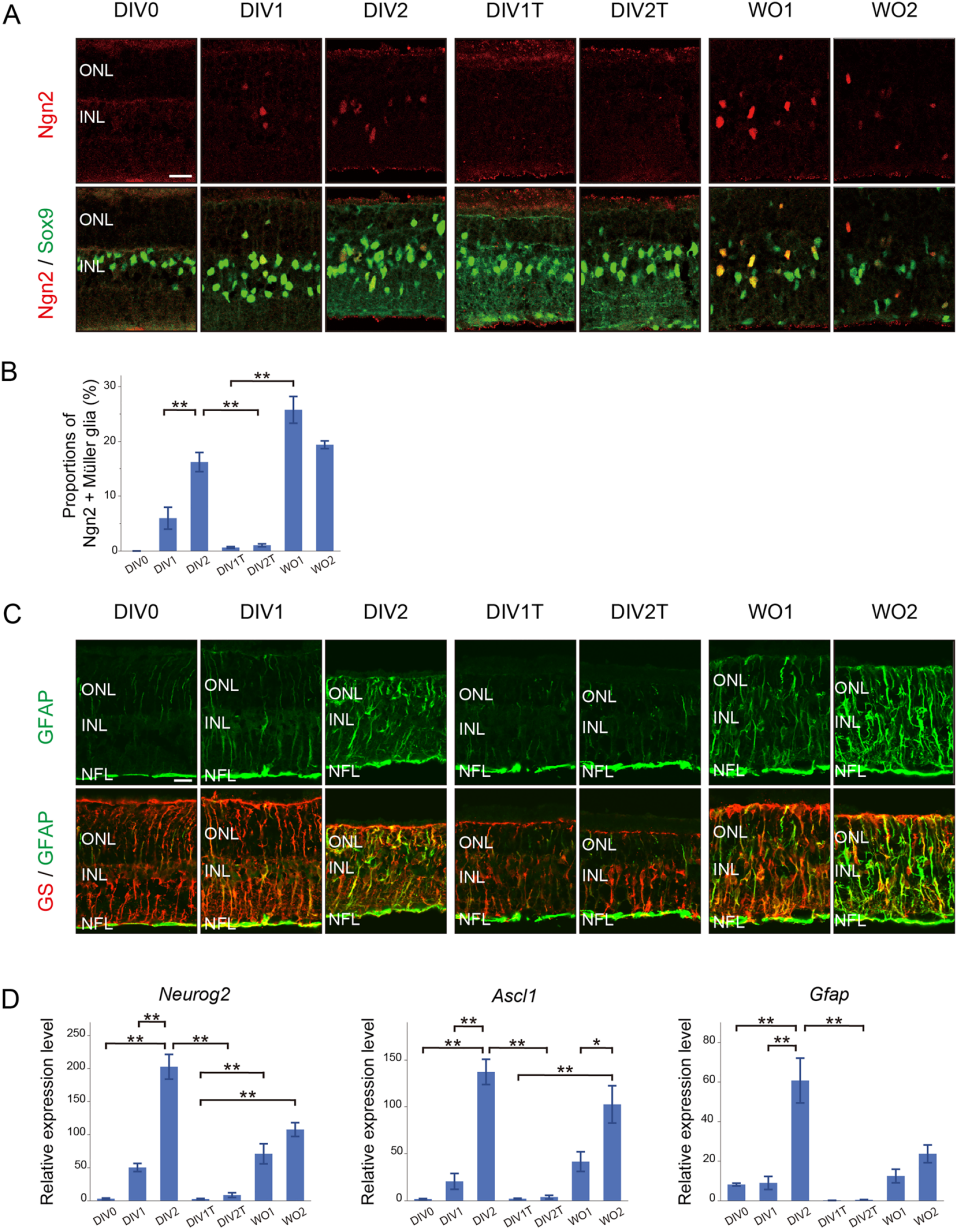
The effects of thymidine-induced cell cycle arrest on the expression of Ngn2, Ascl1 and GFAP in retinal explants after injury. (**A**) Double immunofluorescence for Ngn2 and Sox9. (**B**) Proportions of Ngn2-positive Müller glia. **P < 0.01. (**C**) Double immunofluorescence for GFAP and GS. (**D**) qRT-PCR analyses of *Neurog2*, *Ascl1*, and *Gfap*. DIV, days in vitro; WO, days after wash out; ONL, outer nuclear layer; INL, inner nuclear layer; NFL, nerve fiber layer. Scale bars in (**A**) and (**C**) = 20 µm. Each bar of the graphs represents the mean ± SEM (n = 3). The values in (**D**) were expressed relative to the controls (day0 in vivo) after normalization to *Gapdh* levels. *P < 0.05, **P < 0.01.

We next analyzed the effects of thymidine block on the expression of proneural transcription factors Ngn2 and Ascl1 and a gliosis marker GFAP by immunofluorescence and qRT-PCR. Ngn2-positive Müller glia were not observed at DIV0, but increased in number by DIV2 (corresponding to S phase entry and subsequent cell cycle exit) (Fig. 4A,B), consistent with the timing of Ngn2 expression in vivo (Fig. 1B,C). The proportion of Ngn2-positive Müller glia was approximately 15% at DIV2, which was higher than the values in vivo (Fig. 1C), suggesting that explant culturing may promote reprograming of Müller glia. The number of Ngn2-positive Müller glia was significantly decreased by thymidine treatment (DIV2 vs DIV1T/DIV2T) and increased after release from thymidine block (DIV1T vs WO1/WO2) (Fig. 4A,B). Ascl1 immunoreactivity was not detectable with the antibody used in the present study (data not shown). Immunofluorescence for GFAP/GS revealed an increase in GFAP levels in Müller glia from DIV0 to DIV1/DIV2 (Fig. 4C). This increase was attenuated by thymidine treatment (DIV1T/DIV2T) and release from thymidine treatment notably enhanced GFAP immunoreactivity (WO1/WO2) (Fig. 4C). qRT-PCR analyses confirmed immunofluorescence results. The expression of *Neurog2* (Ngn2), *Ascl1*, and *Gfap* significantly increased from DIV0 to DIV2, and their expression peaked after cell cycle exit of Müller glia (DIV2) (Fig. 4D). The expression of all these genes significantly decreased by thymidine treatment (DIV2 vs DIV1T/DIV2T) (Fig. 4D). The expression of *Neurog2* and *Ascl1* significantly increased by release from thymidine block (DIV1T vs WO1/WO2) and the expression of *Gfap* showed a trend to increase after release (DIV1T vs WO2, *P* = 0.055) (Fig. 4D). We also conducted immunofluorescence for vimentin, a glial filament protein similar to GFAP. Vimentin immunoreactivity in Müller glia did not show apparent changes with and without thymidine treatment (Supplementary Fig. S3).

## Discussion

By using the thymidine block method, we demonstrated that S phase entry of Müller glia promotes their injury-induced migration and expression of proneural factors Ngn2 and Ascl1 as well as a gliosis marker GFAP. To our knowledge, this is the first report of cell cycle-dependent mechanisms regulating injury-induced responses of mammalian Müller glia.

Proneural transcription factors such as Ascl1 and Ngn2 are essential for neuronal fate specification^21^. In the retina, both transcription factors are expressed in retinal progenitors and promote differentiation of retinal neurons while inhibiting glial cell fate^22–25^. In zebrafish, Ascl1 is upregulated in Müller glia after injury and is essential for Müller glia proliferation and neurogenic reprogramming^26,27^. In mammals, both Ascl1 and Ngn2 are induced in immature mouse Müller glia ex vivo^28^, but not in adult mice^3^, indicating age-dependent decline in the reprogramming capacity of mouse Müller glia^4^. In contrast, the present data showed that Ngn2 is induced in adult rat Müller glia after injury both in vivo and ex vivo and that Ascl1 is also induced by injury in the adult rat retina at least ex vivo. Ngn2 was localized to rat Müller glia by immunofluorescence while Ascl1 was not detectable by immunofluorescence. Given the previous reports that Ascl1 is induced in immature mouse Müller glia by Notch inhibition^29^ or growth factor stimulation^28^, it is likely that Ascl1 is also expressed in rat Müller glia. However, further investigations using alternative techniques such as in situ hybridization or cell type-specific qRT-PCR would be required to verify this possibility. Although a substantial fraction of rat Müller glia were shown to express the reprogramming factor Ngn2 after injury, our preliminary observations indicate that only a few Müller glia are capable of expressing neuronal differentiation markers such as Otx2 (Nishino et al. unpublished observation). Thus, actual neurogenesis does not seem to occur in the rat retina after injury.

The species difference in the proneural factor expression in Müller glia may be mechanistically linked to their proliferative ability as rat Müller glia consistently show a proliferative response in a variety of injury models while mouse Müller glia barely proliferate in vivo^14–17^. In zebrafish, Ascl1 is known to promote Müller glia proliferation after injury^26,27^. However, it seems unlikely that Ascl1 and Ngn2 promote Müller glia proliferation in rats as the expression of these factors peaked after Müller glia exited the cell cycle in our retinal injury models. In the embryonic brain, Ascl1 drives cell cycle progression in neuronal progenitors while promoting cell cycle exit in differentiating neurons^30^. Ngn2 is also known to drive cell cycle exit of neuronal progenitors^31,32^. Thus, unlike the situation in zebrafish, injury-induced expression of proneural factors may function to prevent cell cycle progression of Müller glia in rats. On the other hand, our data support the possibility that proliferation plays a critical role in the upregulation of proneural factors in rat Müller glia. Thymidine-induced cell cycle arrest at the G1-S boundary hampered expression of Ascl1 and Ngn2 and release from the arrest stimulated their expression, strongly indicating that the proneural factor expression in rat Müller glia requires S phase entry and progression. Although the mechanism underlying the S phase-dependent activation of proneural genes in Müller glia remains unclear, activation of developmental genes during S phase has been previously reported in other tissues; HoxB gene activation during embryonic development requires DNA replication during S phase^33^ and lineage-specific gene activation during erythroid differentiation also requires S phase^34,35^. Moreover, DNA replication has been shown to promote pluripotency factor expression and reprogramming by stem cell fusion^36^ or somatic cell nuclear transfer^37^. Thus, DNA replication during S phase may provide an opportunity for transcriptional and epigenetic reprogramming as has been suggested previously^36,38^. This notion may also be relevant to the recent observation that cell cycle reentry seems to facilitate neurogenesis of Müller glia stimulated by proneural factor overexpression^39^.

The present data also provide evidence that S phase entry of rat Müller glia promotes their migration to the site of injury. Müller glia migration to the site of injury has been observed in a variety of species^40^, and this migration has been shown to be required for Müller glia proliferation and regeneration in zebrafish^41^. We previously observed species difference in the injury-induced migratory behavior of Müller glia between rats and mice; in rats, many Müller glia migrate into the ONL as they enter S phase after photoreceptor injury while in mice, migration of Müller glia, which remain quiescent after injury, is mostly confined within the INL^16,42^. These observations are in agreement with the possibility that S phase entry triggers Müller glia migration. Although the mechanism linking proliferation and migration of Müller glia remains to be studied, the involvement of signal transduction pathways such as Sphingosine-1-phosphate, PI3K or ERK/MAPK pathways may be an interesting possibility as suggested previously^43^.

The present data that S phase entry of Müller glia activates the expression of GFAP, a hallmark of reactive gliosis, were unexpected, because a previous study in zebrafish reported that Müller glia proliferation promotes neurogenic reprogramming while inhibiting reactive gliosis^13^. Reactive gliosis is considered to hamper neurogenic reprogramming of Müller glia^1^. Thus, in contrast to zebrafish, proliferation of Müller glia may not be simply neurogenic but have antineurogenic effects in mammals. We have previously reported that rat Müller glia exhibit the DNA damage response upon injury-induced cell cycle reentry possibly due to replication stress during S phase^16^. As DNA damage has been shown to induce GFAP upregulation in neural stem cells^44,45^, the DNA damage response may be causally involved in the S phase-associated activation of GFAP in proliferating Müller glia. Alternatively, GFAP expression may be under the control of transcriptional regulators having S phase-dependent expression or function. It is unlikely that proneural factors such as Ascl1 and Ngn2 upregulate GFAP given the previous reports that overexpression of these factors convert mouse Müller glia into cells with neuronal phenotype^7,46^. We have previously reported that gliogenic transcription factor Nfia is upregulated in proliferating mouse Müller glia as they traverse S phase in vitro^17^. As Nfia has been shown to activate GFAP expression in astrocytes^47,48^, upregulation of gliogenic transcription factors such as Nfia in Müller glia may promote GFAP expression in a S phase-dependent manner.

Although cyclin D3 has been established as a marker of quiescent Müller glia^49^, the role of this cell cycle regulator in Müller glia proliferation remains unclear. The present observations that rat Müller glia upregulate GFAP and downregulate cyclin D3 after S phase entry induced by release from thymidine block are in agreement with the previous report that mouse Müller glia upregulate GFAP while downregulating cyclin D3 after injury-induced S phase entry^49^. Our previous study also suggested that Müller glia downregulate cyclin D3 after S phase entry^17^. However, the significant increase in cyclin D3 levels associated with thymidine-induced G1/S arrest of Müller glia in the present study was unexpected. As most Müller glia in mice upregulate cyclin D3 after injury without S phase entry and are considered to be arrested in G1 phase^14,15,17^, similar mechanisms may activate cyclin D3 expression in rat Müller glia arrested in G1 phase by thymidine block.

In conclusion, the present study showed that proliferation of Müller glia promotes neurogenic as well as gliotic responses in adult rat retina. Induction of reactive gliosis by proliferation may be one of the mechanisms underlying the limited regenerative capacity of mammalian Müller glia. Defining how the cell cycle impacts the fate of Müller glia after injury and designing strategies to stimulate Müller glia proliferation without inducing reactive gliosis may be critical to promote retinal regeneration in mammals.

## Methods

### Animals

Male Wistar rats (5 weeks old) were obtained from Charles River Laboratories Japan (Yokohama, Japan). Animals were euthanized with CO_2_ or by decapitation under anesthesia with isoflurane. All animal experiments were conducted according to protocols approved by the institutional animal care committee and to the relevant guidelines and regulations of Tokyo Women’s Medical University. The study was reported in accordance with ARRIVE guidelines.

### Induction of retinal degeneration

Photoreceptor degeneration was induced by a single intraperitoneal injection of N-methyl-N-nitrosourea (MNU, Sigma-Aldrich, St. Louis, MO, USA, 70 mg/kg body weight) as reported previously^16^.

### Retinal explant and cell synchronization

Rat retinas were explanted 2 days after MNU injection as reported previously^18^. The retinas were incubated in 25% Hanks balanced salt solution (Invitrogen, San Diego, CA, USA), 25% heat-inactivated horse serum, 5.75 mg/ml glucose, 200 μM l-glutamine and 50 units/ml penicillin–streptomycin in a humidified atmosphere of 5% CO_2_ at 37 °C. To arrest Müller glia proliferation at the G1/S boundary, explant retinas were cultured in the presence of 30 mM thymidine (FUJIFILM Wako Pure Chemical Corporation, Osaka, Japan). Cell were released from arrest by 2 washes with thymidine-free medium.

### TUNEL assay

Cell death was visualized by TUNEL assay using in situ cell death detection kit, TMR red (Roche, Mannheim, Germany) in accordance with the manufacturer’s instruction.

### Immunofluorescence

The retinas were processed for immunofluorescence as described previously^16,18^. Cryostat Sections (10 μm thick) were incubated with a mixture of primary antibodies overnight and with secondary antibodies for 30 min. Primary and secondary antibodies are listed in Supplementary Table S1. Nuclear counterstaining was performed with 4′,6-diamidino-2-phenylindole (DAPI; Sigma Aldrich). Fluorescence images were obtained by confocal laser scanning microscope (LSM710; Carl Zeiss, Jena, Germany).

### Quantitative RT-PCR

Quantitative RT-PCR (qRT-PCR) was performed as described previously^16,18^. Total RNA was extracted using RNeasy plus kit (QIAGEN, Hilden, Germany) and reverse transcribed using the ReverTra Ace qPCR RT Master Mix with gDNA Remover Kit (Toyobo, Osaka, Japan). qPCR was conducted using Step One Plus and Power SYBR Green PCR Master Mix (Thermo Fisher Scientific). Primers used are shown in the Supplementary Table S1. Data were normalized to *Gapdh* expression and shown as the mean of three samples relative to control levels.

### Cell counting

The central regions (measuring approximately 1300 µm length) or the whole length of the retinas were subjected to cell counting under a fluorescence microscope (DM IL LED; Leica, Tokyo, Japan) as described previously^16,18^. Four sections per animal (three animals per stage or condition) were used for quantitation.

### Statistical analysis

One-way analysis of variance (ANOVA) followed by Tukey–Kramer's post hoc test was conducted using a statistical software (JMP Pro 16). Significance was evaluated at *P* < 0.05.

### Supplementary Information

Supplementary Figure S1.

Supplementary Figure S2.

Supplementary Figure S3.

Supplementary Table S1.

## Data Availability

The datasets generated during the current study are available from the corresponding author on reasonable request.
