# Supplementary material for: Cell cycle-dependent activation of proneural transcription factor expression and reactive gliosis in rat Müller glia
Source: Sci Rep. 2023 Dec 19;13:22712. doi: 10.1038/s41598-023-50222-0 (PMC10733309; doi:10.1038/s41598-023-50222-0)

Supplementary Figure S1. TUNEL assays in the rat retinas after MNU treatment. **A.** TUNEL assays in vivo showing intense labeling in the ONL and subsequent clearance of TUNEL-positive cells. **B.** TUNEL assays combined with Sox9 immunofluorescence in retinal explants with and without thymidine treatment. Higher magnifications of square boxed regions are shown at the bottom. Note TUNEL-positive, DAPI-positive, and Sox9-negative nuclei (arrowheads). **C.** Quantification of TUNEL/DAPI double-positive cells. Each bar represents the mean ± SEM (n = 3). *P<0.05. ONL, outer nuclear layer; INL, inner nuclear layer; GCL, ganglion cell layer. Scale bars in **A** and **B** = 20 μm.

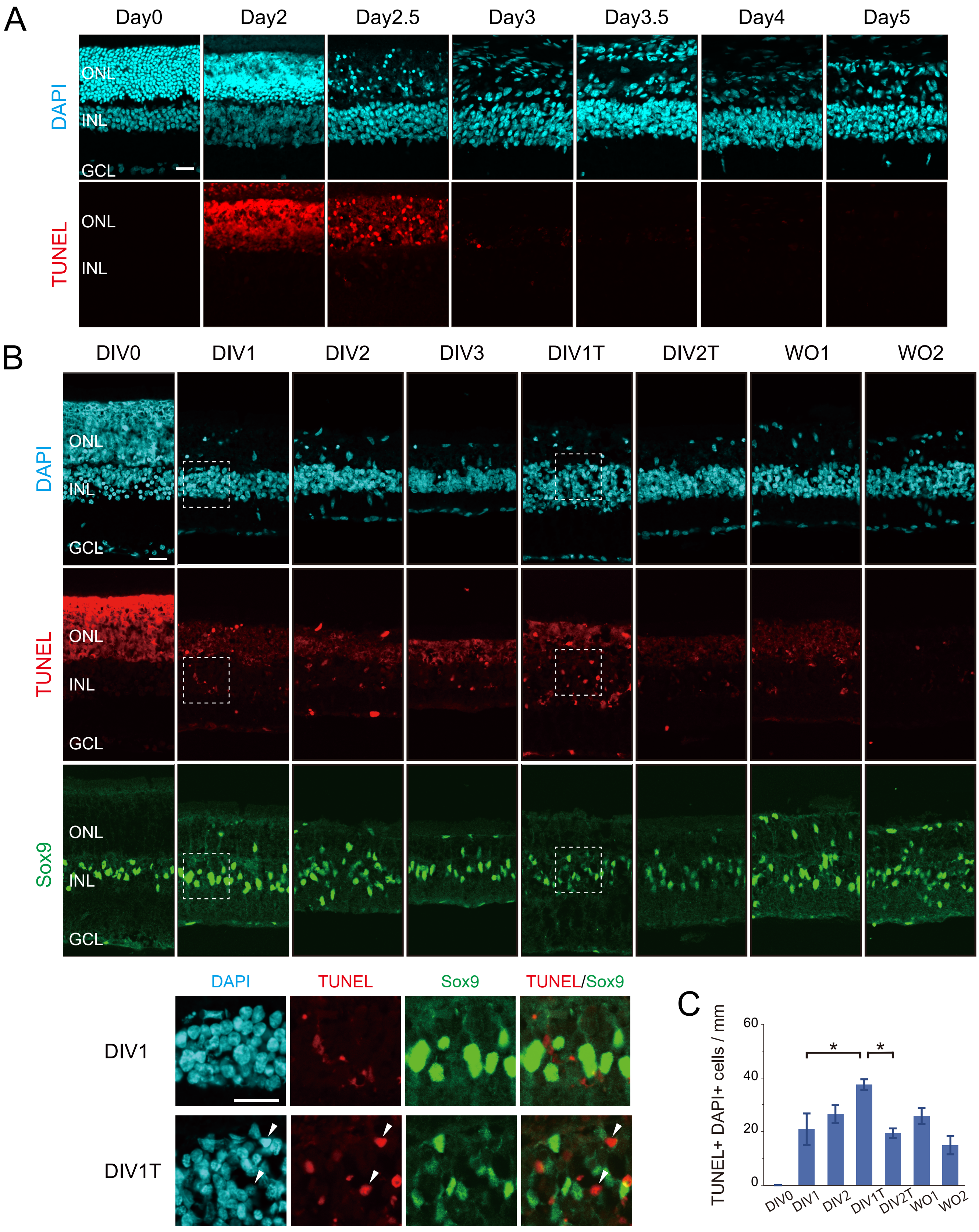

Supplement: Supplementary file 1 — Supplementary Figure S1. [file 41598_2023_50222_MOESM1_ESM.pdf]
