# Supplementary material for: Cell cycle-dependent activation of proneural transcription factor expression and reactive gliosis in rat Müller glia
Source: Sci Rep. 2023 Dec 19;13:22712. doi: 10.1038/s41598-023-50222-0 (PMC10733309; doi:10.1038/s41598-023-50222-0)

Supplementary Figure S2. Immunofluorescence for p27 and cyclin D3 in retinal explants with and without thymidine treatment. **A.** Double immunofluorescence for p27 and Sox9. Higher magnifications of square boxed regions are shown at the bottom. Arrowheads indicate p27-negative Müller glia while arrows denote p27-positive Müller glia. **B.** Double immunofluorescence for cycliln D3 and Sox9. ONL, outer nuclear layer; INL, inner nuclear layer. Scale bar = 20 μm.

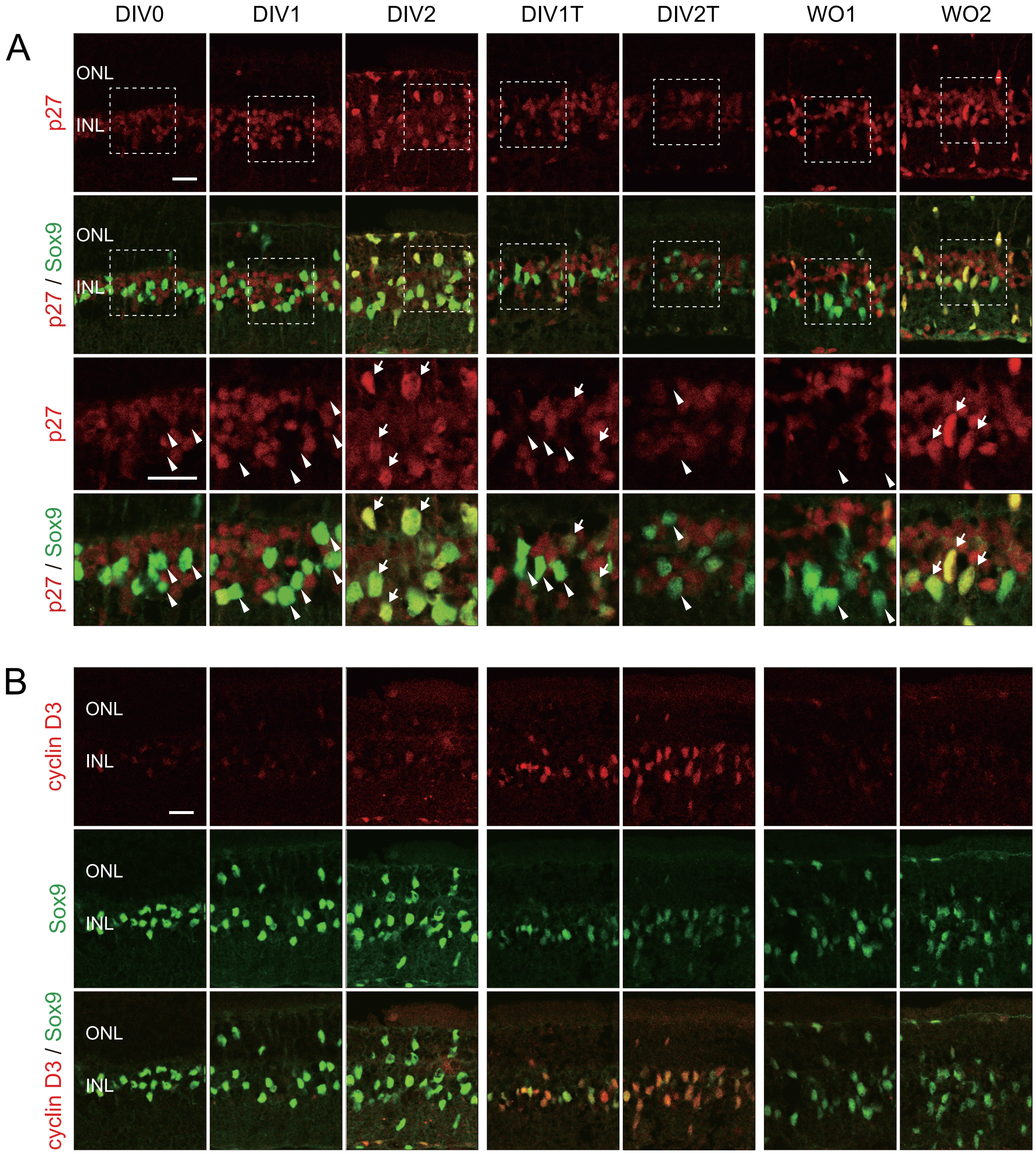

Supplement: Supplementary file 2 — Supplementary Figure S2. [file 41598_2023_50222_MOESM2_ESM.pdf]
