# Supplementary material for: Cell cycle-dependent activation of proneural transcription factor expression and reactive gliosis in rat Müller glia
Source: Sci Rep. 2023 Dec 19;13:22712. doi: 10.1038/s41598-023-50222-0 (PMC10733309; doi:10.1038/s41598-023-50222-0)

Supplementary Figure S3. Immunofluorescence for vimentin and glutamine synthetase (GS) in retinal explants with and without thymidine treatment. ONL, outer nuclear layer; INL, inner nuclear layer; GCL, ganglion cell layer. Scale bar = 20 μm.

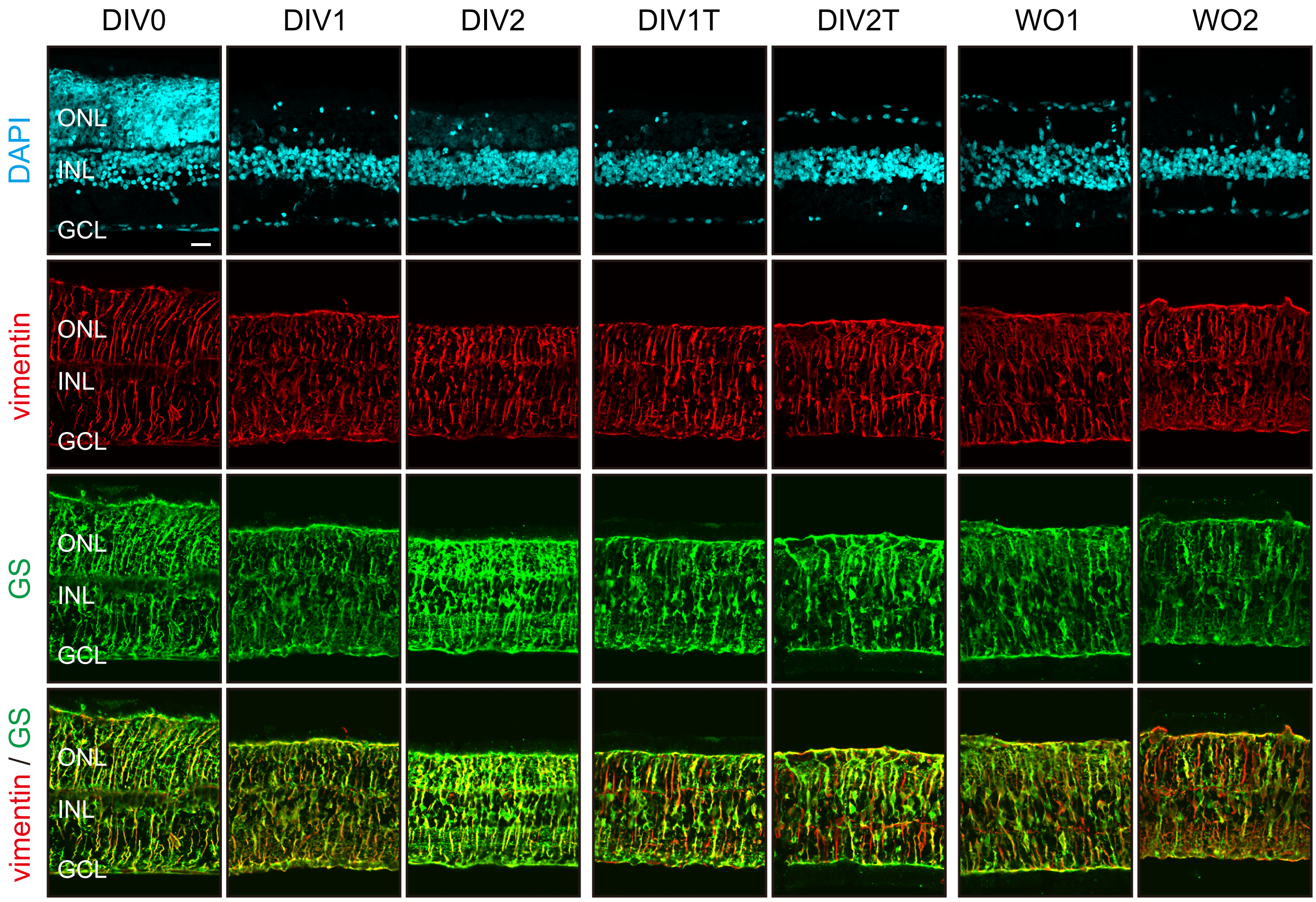

Supplement: Supplementary file 3 — Supplementary Figure S3. [file 41598_2023_50222_MOESM3_ESM.pdf]
