# Supplementary material for: Cell cycle-dependent activation of proneural transcription factor expression and reactive gliosis in rat Müller glia
Source: Sci Rep. 2023 Dec 19;13:22712. doi: 10.1038/s41598-023-50222-0 (PMC10733309; doi:10.1038/s41598-023-50222-0)
Supplement: Supplementary file 4 — Supplementary Table S1. [file 41598_2023_50222_MOESM4_ESM.pdf]

Supplementary Table S1.

List of primary antibodies

Antibody	Dilution	Species	Source
Cdc2	1:500	Mouse	Santa cruz sc-54
Cyclin D3	1:1000	Mouse	Cell Signaling 2936S
GFAP	1:5000	Guinea pig	Synaptic systems 173 004
Glutamine synthetase (GS)	1:5000	Rabbit	Sigma-Aldrich G2781-100UL
Glutamine synthetase (GS)	1:1000	Mouse	Millipore MAB302
Lhx2	1:2000	Mouse	Atlas AMAb91404
Neurogenin2	1:1000	Goat	Santa cruz sc-19233
Phospho-histone H3	1:10000	Mouse	Upstate 05-800
Phospho-pRb	1:1000	Rabbit	Cell Signaling 8516
Sox9	1:2000	Rabbit	Millipore AB5535
Sox9	1:2000	Goat	RD AF3075
Vimentin	1:900	Rabbit	Abcam ab92547
$p27^{kip1}$	1:1000	Mouse	BD 610241

List of secondary antibodies

Antibody	Dilution	Source
Donkey anti-rabbit IgG Alexa Fluor 555	1:1000	Invitrogen A-31572
Donkey anti-rabbit IgG Alexa Fluor 488	1:1000	Invitrogen A-21206
Donkey anti-mouse IgG Alexa Fluor 647	1:1000	Invitrogen A-31571
Donkey anti-mouse IgG Alexa Fluor 555	1:1000	Invitrogen A-31570
Donkey anti-mouse IgG Alexa Fluor 488	1:1000	Invitrogen A-21202
Donkey anti-goat IgG Alexa Fluor 555	1:1000	Invitrogen A-21432
Donkey anti-goat IgG Alexa Fluor 488	1:1000	Invitrogen A-11055
Goat anti-guinea pig IgG Alexa Fluor 488	1:1000	Invitrogen A-11073

List of primers

Gene	Forward	Reverse
Gapdh	ACAAGATGGTGAAGGTCGGTGTGA	AGCTTCCCATTCTCAGCCTTGACT
Ccnd1	CAGAAGTGCGAAGAGGAGGTC	TCATCTTAGAGGCCACGAACAT
Ccne1	GAAAAGCCAGGATAGCAGTCAG	CCCCAATTCAAGACGGGAAG
Ccne2	AGGAATCAGTCCTTGCATTATC	CCCAGCTTAAGTCTGGCAGAG
Ccna2	GCCTTCACCATTCATGTGGAT	TGGCTCCGGGTAAAGAGACAG
Neurog2	GTCCTCCTCCAACTCCAC	GTGCATAACGGTGCTTCT
Ascl1	GAGGGATCCTACGACCCCCTTAGTC	CTCCTGCCATCCTGCTTCCAAAG
Gfap	CGGAGACGTATCACCTCTG	AGGGAGTGGAGGCGTCATTCG
